# An Unusual Abdominal Tumor—Case Report

**Published:** 1917-05

**Authors:** M. Smith

**Affiliations:** Oklahoma City, Okla.


### An Unusual Abdominal Tumor—Case Report.

BY DR. M. SMITH, OKLAHOMA CITY, OKLA.

This case came under my observation July 20, 1916. General appearance was that of marked emaciation with secondary anemia. Temperature 100, pulse 100, tongue heavily coated, nauseated, marked dyspnea. Heart, lungs and liver normal. Diarrhea with small amount of blood or mucus tinged with blood in the stools. There were from four to five movements in twenty-four hours, attended with pain and griping. Stool was liquid and very offensive. The abdomen was very greatly distended and dull upon percussion, the dullness extending from ensiform cartilage to symphysis and laterally to each flank. A change of position produced no change in the percussion note.

There were areas over the abdomen over which the note was flat rather than dull, though these areas could not be definitely outlined. Vaginal examination elicited nothing except slight bulging in cul-de-sac with cystocele and rectocele.

Upon palpation, an indistinct tumor mass could be felt filling the entire peritoneal cavity, immovable and smooth.

No pain was elicited upon deep pressure but rather a sense of discomfort.

*History.*—The history of the case prior to coming under my care is as follows:

Family history, negative. Married at age of 17, menstrual period began at the age of 14, regular and normal in every respect. Has given birth to four children, all of which labors were normal. The youngest of her children was two years old at time of her admission to hospital. One year prior to this time she aborted from no known cause. The last child died at three and one-half months of marasmus. Her personal history is that of good health up until the birth of her last child, following which she noticed that her abdomen never returned to normal size or shape, and she could herself outline a tumor mass. At this time she had pains in the side and abdomen, which she described as cramping in character, and she was, as a rule, nauseated during these attacks.

Patient placed upon table July 24th, 10 a. m.; incision made from umbilicus to symphysis pubes.

After getting through what appeared to be the peritoneum, we came to the tumor proper. The wound retracted, there appeared a thick, glistening membrane evidently covering the entire peritoneal cavity.

Upon opening this sac a quantity of a greenish-tinted fluid mixed with large masses of gelatinous substance escaped. All the fluid and gelatinous material possible having been removed, I attempted to remove the tumor, which I then thought a large multilocular cyst with extensive parietal adhesions.

This I found impossible. A piece was sent to the laboratory for quick diagnosis. The report was soon returned that it was tubercular. However, I was not satisfied with this report.

A most interesting point during operation was that during all my exploration I did not see an intestine nor an outline of an intestine, nor could they be made out through the posterior cyst wall.

However, the fundus of the uterus could be outlined, and both tubes were removed without ligation, the tissue being so degenerated that hemostasis by ligation was impossible and gauze packing was resorted to to control the bleeding.

At this time patient began to show evidence of decided shock, the abdomen was closed with through and through silkworm stitches and the patient put to bed, the usual treatment being resorted to.

She reacted nicely, and on the following day her condition was very satisfactory.

The wound healed by first intention except at the site of drainage. The drainage was rather profuse and purulent in character, continuing until the present time, though gradually lessening in amount.

She is at present able to care for her household duties, and her weight is normal.

Around the site of drainage a hard mass can be outlined.

POINTS OF INTEREST.

1. Its development during pregnancy without interfering with pregnancy.

2. Was it tubercular peritonitis or was it a tubercular cystic degeneration, if such could occur?

3. Where were the intestines?

